# „Youth Mental Health“: Früherkennung und Frühintervention psychischer Erkrankungen im Jugendalter

**DOI:** 10.1007/s00115-025-01847-1

**Published:** 2025-06-25

**Authors:** Peter J. Uhlhaas, Andreas Bechdolf, Kerstin Konrad, Nikolaos Koutsouleris, Eva Meisenzahl, Andreas Meyer-Lindenberg, Ulrich Reininghaus, Christoph U. Correll

**Affiliations:** 1 Department of Child and Adolescent Psychiatry, Charité-Universitätsmedizin, Berlin, Deutschland; 2 German Center for Mental Health (DZPG), partner site Berlin-Potsdam, Berlin, Deutschland; 3 Department of Psychiatry, Psychotherapy und Psychosomatic, Vivantes Klinikum am Urban und Vivantes Klinikum im Friedrichshain, Berlin, Deutschland; 4 Department of Psychiatry and Psychotherapy, CCM, Charité – Universitätsmedizin, Berlin, Deutschland; 5 Child Neuropsychology Section, Department of Child and Adolescent Psychiatry, RWTH, Aachen, Deutschland; 6 JARA-Brain Institute II, Molecular Neuroscience and Neuroimaging, Research Center Jülich, Aachen, Deutschland; 7 Department of Psychiatry and Psychotherapy, Ludwig-Maximilians-University, München, Deutschland; 8 German Center for Mental Health (DZPG), partner site Munich-Augsburg, Munich, Deutschland; 9 Department of Psychiatry and Psychotherapy, Medical Faculty, Heinrich-Heine University, LVR Düsseldorf, Düsseldorf, Deutschland; 10 Central Institute of Mental Health, Medical Faculty Mannheim, Heidelberg University, Mannheim, Deutschland; 11 German Center for Mental Health (DZPG), partner site Mannheim-Heidelberg-Ulm, Mannheim, Deutschland; 12 Department of Public Mental Health, Central Institute of Mental Health, Medical Faculty Mannheim, Heidelberg University, Mannheim, Deutschland; 13 ESRC Centre for Society and Mental Health, King’s College London, London, Großbritannien; 14 Departments of Psychiatry and Molecular Medicine, Donald and Barbara Zucker School of Medicine at Hostra/Northwell, Hempstead, USA; 15 Department of Psychiatry, The Zucker Hillside Hospital, Northwell Health, Glen Oaks, NY USA

**Keywords:** Youth Mental Health, Früherkennung, Frühintervention, Sensitive Phasen, Jugendalter, Youth mental health, Early intervention, Early detection, Sensitive phases, Adolescence

## Abstract

**Hintergrund:**

Psychische Erkrankungen treten besonders häufig zwischen dem 12. und 25. Lebensjahr auf, mit wichtigen Implikationen für die Pathogenese, Diagnose und Behandlung.

**Ziel der Arbeit:**

Vor diesem Hintergrund skizzieren wir in diesem Artikel ein „Youth-Mental-Health“-Paradigma, in dem die Früherkennung, Frühintervention und Prävention psychischer Erkrankungen, wie z. B. Psychosen, bipolare Störungen und Persönlichkeitsstörungen, im Jugendalter im Mittelpunkt steht.

**Material und Methoden:**

Im ersten Teil des Artikels fassen wir Befunde zu „sensitiven Phasen“ in der Gehirnentwicklung im Jugendalter zusammen, die auf die besondere Plastizität neuronaler Schaltkreise hindeuten, die für präventive Interventionen genutzt werden können. Die Wechselwirkungen mit Risiko- und Resilienzfaktoren im Jugendalter sind ein wesentlicher Grund für das häufige Auftreten psychischer Erkrankungen. Des Weiteren werden innovative diagnostische Ansätze, wie z. B. „klinische Stadienmodelle“, vorgeschlagen, die eine Voraussetzung für Früherkennung und Frühintervention im Jugendalter sind. Hier sind niedrigschwellige Versorgungsstrukturen sowie altersgerechte Interventionen, z. B. durch digitale Ansätze, erforderlich, um Jugendlichen einen besseren Zugang zu klinischen Angeboten zu ermöglichen.

**Diskussion:**

Die Früherkennung und Frühintervention bei psychischen Erkrankungen stellen möglicherweise einen wichtigen Ansatz für die Forschung und Klinik dar, um langfristig die psychische Gesundheit bei Jugendlichen und jungen Erwachsenen zu verbessern.

## Abstract (en)

**Background:**

Mental diseases occur particularly frequently between the ages of 12 and 25 years, with important implications for the pathogenesis, diagnosis and treatment.

**Objective:**

Against this background this article outlines a youth mental health paradigm which prioritizes the early detection, early intervention and prevention of mental disorders, e.g., psychoses, bipolar disorders and personality disorders in adolescence.

**Material and methods:**

The first part of the article summarizes the findings for sensitive phases during brain development, which indicate the elevated plasticity of neural circuits during youth that can be used for preventive interventions. The interactions between risk and resilience factors and ongoing brain development are an essential reason for the frequent occurrence of mental disorders between 12–25 years. Furthermore, innovative diagnostic approaches, such as clinical staging models, are proposed that are a prerequisite for early detection and early intervention. In this context, low-threshold service modelss and age-appropriate interventions, such as digital approaches, are necessary to improve access to care in youths.

**Discussion:**

Taken together, a “youth mental health” paradigm could significantly enhance mental health in young people and initiate a shift towards the prevention of severe mental disorders.

## Hintergrund

**Abb. 1:**
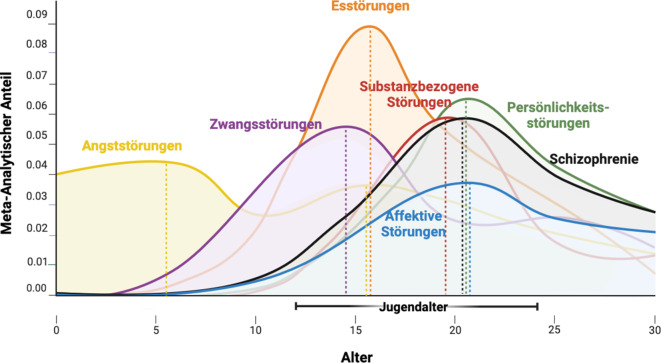
Entwicklung und Auftreten psychischer Erkrankungen. Alter und Beginn psychischer Erkrankungen in der Allgemeinbevölkerung auf der Grundlage der Metaanalyse von Solmi et al. [3]: metaanalytischer epidemiologischer Anteil (y-Achse) für Angststörungen (5,5/15,5 Jahre), substanzbezogene Störungen (19,5 Jahre), Schizophrenie/Psychosen (20,5 Jahre), Essstörungen (15,5 Jahre), Persönlichkeitsstörungen (20,5 Jahre), Zwangsstörungen (14,5) und affektive Störungen (20,5 Jahre; ICD-10-Blöcke [ICD: International Statistical Classification of Diseases and Related Health Problems]). Die *gestrichelten* *Linien* stellen die höchste Inzidenz für jede Diagnosekategorie dar

Psychische Erkrankungen stellen weiterhin für die Gesellschaft wie auch für die Forschung eine große Herausforderung dar [1]. Bislang stand die Behandlung voll ausgebildeter Störungsbilder im Erwachsenenalter im Vordergrund, während Frühintervention und Prävention vernachlässigt wurden [2]. Inzwischen gibt es jedoch übereinstimmende epidemiologische Daten, dass psychische Erkrankungen, wie z. B. Psychosen, affektive Störungen und Persönlichkeitsstörungen, besonders häufig im Jugendalter, d. h. zwischen dem 12. und 25. Lebensjahr, auftreten ([3]; Abb. 1). Gleichzeitig haben Jugendliche und junge Erwachsene schlechten Zugang zu klinischen Angeboten [4].

In diesem Artikel schlagen wir daher ein transformatives Paradigma für die Psychiatrie vor, dessen Fokus auf der Frühintervention, Früherkennung und Prävention psychischer Störungen im Jugendalter gerichtet ist. Im ersten Teil fassen wir aktuelle Daten zur Entwicklung kognitiver Funktionen und der zugrunde liegenden neuronalen Schaltkreise im Jugendalter zusammen, die auf „sensitive Phasen“ in der Gehirnentwicklung hindeuten und wichtige Zeitfenster für die Frühintervention und Prävention darstellen könnten. Im zweiten Teil diskutieren wir die besonderen Anforderungen für diagnostische Ansätze zu Früherkennung und Frühintervention. Zuletzt werden die Entwicklungen im Bereich von Versorgungsmodellen sowie die Bedeutung digitaler Interventionen für Jugendliche und junge Erwachsene dargestellt.

## Gehirnentwicklung, sensitive Phasen und Psychopathologie

**Abb. 2:**
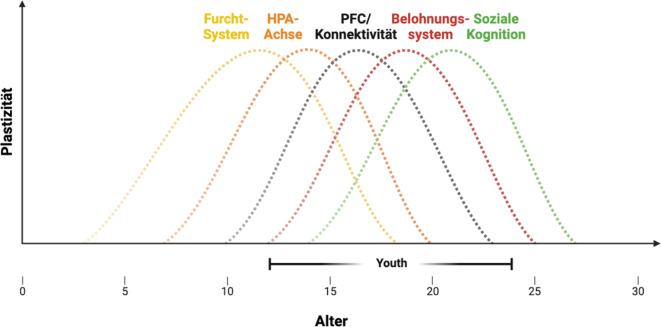
Sensitive Phasen und Gehirnentwicklung. Die Kurven zeigen das plastische Potenzial für verschiedene neuronale Systeme: (*gelb*) Furchtregulation unter Einbeziehung kortikal-hippokampaler Amygdalaschaltkreise, (*orange*) HPA-Achsen-System, (*schwarz*) PFC/Konnektivität mit lokalen Veränderungen der PFCs (E/I-Balance, Dopamin) sowie weitreichende Konnektivität mit kortikal-subkortikalen Zielregionen, (*rot*) Belohnungssystem umfasst Striatum und Konnektivität mit PFC und (*grün*) sozial-kognitive Prozesse. *HPA-Achse* Hypothalamus-Hypophysen-Nebennieren-Achse, *PFC* präfrontaler Kortex, *E/I-Balance* Erregungs/Hemmungs-Gleichgewicht, *PFC* präfrontaler Kortex

Das Auftreten psychischer Erkrankungen im Jugendalter überschneidet sich mit tiefgreifenden Veränderungen in der Architektur und Funktionsweise neuronaler Schaltkreise. „Sensitive Phasen“ definieren zeitlich begrenzte Entwicklungsfenster, in denen Umwelteinflüsse aufgrund erhöhter Plastizität eine nachhaltige Wirkung auf die Funktionalität und Organisation neuronaler Schaltkreise und Verhalten haben [5]. Ursprünglich wurden sensitive Phasen für frühe Entwicklungsperioden beschrieben, insbesondere für das visuelle System. Inzwischen gibt es konvergierende Befunde, dass neuronale Schaltkreise, die für die Entstehung von Psychopathologie im Jugendalter zentral sind, Kriterien für sensitive Phasen erfüllen (Abb. 2).

Angststörungen treten häufig in den ersten Lebensjahren sowie im Jugendalter auf [9]. Neuere Studien haben zentrale Veränderungen in den neuronalen Schaltkreisen der Furchtregulation während des Jugendalters untersucht [6]. So ist z. B. die Furchtlöschung während der Adoleszenz im Vergleich zur Kindheit und zum Erwachsenenalter reduziert [7], welche mit veränderter synaptischer Plastizität und Konnektivität des präfrontalen Kortex und Verbindungen mit dem Hippokampus und der Amygdala einhergeht [7].

Die Hypothalamus-Hypophysen-Nebennieren-Achse (HPA-Achse) reguliert die Stressreaktion durch die Freisetzung von Glukokortikoiden, und eine Dysregulation der HPA-Achse trägt zur Entstehung von Psychopathologie bei [8]. Tierexperimentelle Studien deuten darauf hin, dass eine Reihe von Stressoren mit erhöhten und verlängerten HPA-Reaktionen im Jugendalter verbunden sind und dass Unterschiede in psychopathologischen Phänotypen vom Zeitpunkt der Stressbelastung abhängen können [9]. Umgekehrt kann die Anreicherung der Umwelt bei adoleszenten Tieren die Auswirkungen pränatalen Stresses und mütterlicher Trennung umkehren [10].

Veränderungen in höheren kognitiven Funktionen, wie z. B. Arbeitsgedächtnis (WM), Inhibition sowie kognitive Kontrolle, sind ein zentraler Aspekt der Gehirnentwicklung im Jugendalter [11]. Anatomisch stehen diese Funktionen in engem Zusammenhang mit der Modifikation präfrontaler Schaltkreise (PFC), z. B. durch die Veränderungen in der Zusammensetzung und Interaktion der dopaminergen, glutamatergen und GABAergen Rezeptoren. Parvalbuminpositive (PV^+^)-Interneurone sind von besonderem Interesse, da diese zum Öffnen und Schließen sensitiver Phasen beitragen [5]. Während des Jugendalters nimmt die Expression von PV^+^-Interneuronen im PFC zu, während eine zeitlich begrenzte Dysregulation zu dauerhaften Veränderungen der Balance zwischen Exzitation und Inhibition (E/I-Balance) im Erwachsenenalter führt [12], ein Prozess, der möglicherweise an der Entwicklung von Psychosen beteiligt ist.

Die Veränderungen der lokalen Schaltkreise des PFC gehen einher mit der umfassenden Integration kortikalen und subkortikalen Areale durch die Reifung weitreichender Verbindungen [13]. Die Verbesserung exekutiver Kontrolle durch präfrontale Regionen spiegelt dabei eine mögliche neurobiologische Grundlage für Verbesserungen bei der Emotionsregulation wider, die bei affektiven Störungen [14], aber auch bei Borderline-Persönlichkeitsstörungen [15] stark beeinträchtigt ist.

Eine dysregulierte limbisch-präfrontale Kommunikation im Jugendalter ist des Weiteren mit Risikoverhalten [16], wie z. B. Substanzabusus, in Verbindung gebracht worden. Die Wahrscheinlichkeit, eine Substanzabhängigkeit zu entwickeln, ist am höchsten, wenn der Missbrauch vor dem 14. Lebensjahr beginnt [17] und kann zu dauerhaften Beeinträchtigungen im Verhalten sowie in der Architektur des Belohnungssystems führen. Tetrahydrocannabinol (THC), der wichtigste psychoaktive Bestandteil von Cannabis, verändert nachhaltig die E/I-Balance und kognitive Funktionen im Jugendalter [18]. Im Zusammenhang mit der Legalisierung von Cannabis ist es daher von besonderer Dringlichkeit, die Auswirkungen auf die Gehirnentwicklung genauer zu erforschen.

Schließlich haben mehrere Studien die Möglichkeit einer sensitiven Phase für die Entwicklung der sozialen Kognition im Jugendalter diskutiert [19]. Das Belohnungssystem reagiert bei Jugendlichen z. B. besonders auf den Einfluss von Gleichaltrigen, was mit einer höheren Risikobereitschaft im sozialen Umfeld zusammenhängen könnte [20]. Soziale Interaktionen im Jugendalter sind auch eine notwendige Voraussetzung für die Entwicklung neuronaler Schaltkreise im PFC [21].

## Psychopathologie und Diagnostik im Jugendalter

Der Beginn der meisten psychischen Störungen im Jugendalter [3] hat wichtige Implikationen für die Diagnose von psychischen Erkrankungen. Die derzeitigen Klassifikationssysteme (DSM‑5, ICD-11) sind jedoch für die Früherkennung und Frühintervention weniger geeignet, da eine Diagnose und der damit verbundene Beginn einer Behandlung nur dann in der Regel erfolgt, wenn die Symptomausprägung über einem bestimmten Cut-off-Wert liegt. Mittlerweile gibt es jedoch Evidenz für Prodromalphasen bei Psychosen [22] und bipolaren Störungen [23] sowie möglicherweise für Essstörungen [24], Depressionen [25] und Zwangsstörungen [26], die alternative diagnostische Ansätze erfordern, um Früherkennung und Frühinterventionen zu ermöglichen.

Hochrisikokriterien für Psychosen („clinical high-risk criteria for psychosis“, CHR-P) wurden erstmals vor über 20 Jahren entwickelt [22] und umfassen abgeschwächte psychotische Symptome (APS), intermittierende psychotische Symptome (BLIPS), ein genetisches Risiko verbunden mit Funktionseinbußen (GRD) sowie Basissymptome, die durch selbsterlebte kognitive und perzeptuelle Defizite definiert werden [27]. CHR-P-Kriterien sind mit einer hohen prognostischen Genauigkeit verbunden [28] und ca. 20 % der Betroffenen entwickeln eine psychotische Ersterkrankung (FEP) in den ersten zwei Jahren [22], deren Prädiktion durch klinische [29], kognitive [30] und bildgebende Verfahren [31] verbessert werden kann.

In Anlehnung an das CHR-P-Konzept wurden Hochrisikokriterien für bipolare Störungen (CHR-BP) entwickelt, die abgeschwächte Formen der Manie und depressive Symptome umfassen [32]. Erste Studien deuten darauf hin, dass CHR-BP-Kriterien mit einer Konversionsrate zu einer bipolaren Störung von 14,3 % innerhalb von 12 Monaten und bis zu 23,4 % nach zehn Jahren verbunden sind [33, 34].

Aufbauend auf den Hochrisikokriterien für Psychosen und bipolare Störungen (CHR-P/BP) wurden dimensionale Konzepte für die Früherkennung entwickelt. Das „klinische Stadienmodell“ positioniert Personen entlang eines multidimensionalen Gradienten von Gesundheit bis Krankheit, der Risikofaktoren, sowie Beginn, Verlauf und Entwicklung der Krankheit erfasst [35]. Ähnlich wie das klinische Stadienmodell in anderen Bereichen der Medizin bietet dieser Ansatz einen Rahmen für präventive Interventionen, wobei weniger intensive Verfahren in früheren Stadien bevorzugt werden und Interventionen mit einem höheren Risiko-Nutzen-Verhältnis späteren Krankheitsstadien vorbehalten sind. Klinische Stadienmodelle für psychische Erkrankungen bei Jugendlichen und jungen Erwachsenen wurden in den letzten Jahren detailliert beschrieben, wobei sich einige an spezifischen diagnostischen Kategorien orientieren, während andere transdiagnostischer Natur sind [36].

Für die Prävention psychischer Erkrankungen sind jedoch weitergehende Ansätze für die Früherkennung und Frühintervention erforderlich, insbesondere für die primäre Prävention. Hierzu bieten Entwicklungen in der normativen Modellierung neue Möglichkeiten, anhand großer Datensätze Abweichungen in zentralen Entwicklungsparametern zu identifizieren [37]. Eine wichtige Voraussetzung ist dabei die Anwendung von „Wachstumskurven“, welche auf normativen Entwicklungsdaten beruhen, die wichtige Bereiche der Psychopathologie und Bildgebungsdaten (fMRI/MRI) umfassen sowie kognitive Funktionen, Emotionsregulation und Schlaf.

## Interventionen und Versorgungsmodelle

Die Befunde zu sensitiven Phasen sowie zur Inzidenz psychischer Störungen im Jugendalter haben wichtige Konsequenzen für die klinische Versorgung und Behandlungsmodelle. Die derzeitigen Versorgungsstrukturen orientieren sich jedoch nach wie vor an etablierten Diagnosekategorien für psychische Störungen im Erwachsenenalter [2]. Daher sind neue Ansätze für die psychische Gesundheit von Jugendlichen und jungen Erwachsenen erforderlich, die den Schwerpunkt auf Frühintervention, niedrigschwellige Versorgungsstrukturen und Prävention legen.

### Sensitive Phasen und Interventionen

**Abb. 3:**
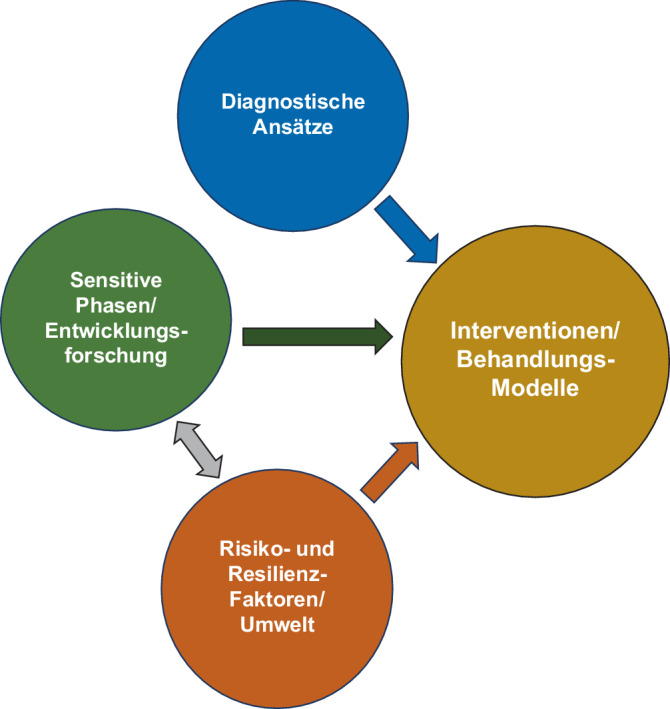
Interaktionen der verschiedenen „Säulen“ des Youth-Mental-Health-Paradigmas bestehend aus Entwicklungsforschung, Risiko und Resilienzfaktoren, diagnostischen Ansätzen und Interventionen/Service-Modellen: Befunde aus der Entwicklungsforschung zu sensitiven Phasen sind relevant für die Identifizierung neuer Behandlungsansätze und Implementierung präventiver Interventionen. Die Synthese und Integration von umweltbasierten Risiko- und Resilienzfaktoren und Entwicklungsdaten zu sensitiven Phasen ist sowohl zentral für mechanistische Krankheitsmodelle als auch für klinische Interventionen. Um niedrigschwellige und transdiagnostische Interventionen und Service-Modelle für Jugendliche und junge Erwachsene zu implementieren, sind innovative diagnostische Ansätze notwendig

Die Befunde zu sensitiven Phasen im Jugendalter könnten demnach wichtige Zeitfenster für Interventionen darstellen, um Entwicklungsprozesse nachhaltig zu beeinflussen (Abb. 3). Derzeit ist der Behandlungsbeginn bei den meisten Störungsbildern, wie z. B. Psychosen, bipolaren Störungen und Essstörungen, mit erheblichen, mehrjährigen Verzögerungen nach dem Auftreten der Symptomatik verbunden [38–40].

Die Dauer der unbehandelten Psychose (DUP) ist eine wichtige Determinante für die symptomatische und funktionelle „Response“ auf die Behandlung bei FEP-Patienten [20]. Ähnliche Daten liegen für die Dauer der unbehandelten Erkrankung (DUI) bei Borderline-Persönlichkeitsstörungen [41], Zwangsstörungen [26] und Essstörungen [42] vor. Darüber hinaus gibt es Hinweise aus der indizierten Prävention bei FEP-Patienten [43], dass spezialisierte psychosoziale und pharmakologische Behandlungsangebote den klinischen Verlauf im Vergleich zur Standardbehandlung verbessern können.

Die Bedeutung sensitiver Phasen für die Frühintervention wird nicht zuletzt durch Studien zu Tiermodellen der Schizophrenie unterstützt, die zeigen, dass verhaltensorientierte und neurobiologische Interventionen in der Adoleszenz, nicht aber in früheren oder späteren Entwicklungsphasen, kognitive Defizite und neuronale Funktionsstörungen vollständig beheben können [44, 45].

### Youth Mental Health und Versorgungsmodelle

In den letzten Jahren wurden neue klinische Versorgungsmodelle für Jugendliche und junge Erwachsene entwickelt, die u. a. die besondere soziokulturelle Einbettung dieser Altersgruppe berücksichtigt. Das erste und inzwischen umfangreichste Beispiel ist das australische Headspace-Programm, ein landesweites Behandlungsmodell für Jugendliche und junge Erwachsene, das darauf abzielt, niedrigschwellige klinische Angebote bei psychischen Störungen und Substanzkonsum bereitzustellen [46]. Behandlungsmodelle, die sich am australischen Headspace-Programm orientieren, werden mittlerweile in vielen Ländern implementiert [47] und mit dem „Soulspace“ in Berlin findet sich ein erstes Beispiel in Deutschland [48].

### E-Mental Health

Früherkennung und Frühinterintervention im Jugendalter könnten von internetgestützten Interventionen und digitalen Technologien profitieren. Lettie et al. [49] weisen jedoch darauf hin, dass momentan nur wenige E‑Mental-Health-Studien, die digitale Ansätze und Technologien für die psychische Gesundheit von Jugendlichen und jungen Erwachsenen evaluieren, in realen klinischen Umgebungen untersucht bzw. repliziert wurden. Darüber hinaus ist die Wirksamkeit der derzeit verfügbaren kommerziellen Apps für psychische Gesundheit bei Jugendlichen und jungen Erwachsenen insgesamt gar nicht bzw. nicht ausreichend wissenschaftlich belegt [43].

„Hybride“ Modelle kombinieren die digitale Intervention mit menschlicher Unterstützung wie z. B. die moderierte Online-Sozialtherapie (MOST; [50]). Ein weiteres Beispiel ist die EMIcompass-App, die digitale Signale aus dem täglichen Leben (z. B. Schlafmuster, Stimmung) erfasst und diese Daten nutzt, um personalisierte und zeitnahe Interventionen anzubieten [51].

E‑Mental-Health-Ansätze könnten auch für die Diagnose von beginnenden psychischen Erkrankungen im Jugendalter in der Allgemeinbevölkerung eingesetzt werden – eine wichtige Voraussetzung für bevölkerungsbasierte Präventionsansätze. In einer aktuellen Studie ermöglichte eine webbasierte Screeningplattform die Identifizierung von Jugendlichen mit einer beginnenden psychotischen Störung mit guter Sensitivität und Spezifität [52]. App-basierte Verfahren könnten auch die Vorhersage von Rückfällen bei Patienten mit Psychosen aufgrund von Symptomen und Verhaltensmustern verbessern [53].

### Partizipative Entwicklung und Beforschung bei Youth Mental Health

Die Entwicklung neuer Interventionen und Behandlungsansätze sowie Forschungsfragen erfordert die Berücksichtigung der einzigartigen Lebenswelten von Jugendlichen und jungen Erwachsenen. Konkret schlagen wir einen bidirektionalen Wissensaustausch zwischen Forschern, Klinikern und Jugendlichen mit gelebter Erfahrung vor [54]. Patient und Public Involvement (PPI) wird in den letzten Jahren verstärkt in Gesundheits- und Forschungsstrukturen für psychischen Erkrankungen integriert [55]. Die Beteiligung von Jugendlichen und jungen Erwachsenen ist daher von entscheidender Bedeutung um sicherzustellen, dass z. B. die angebotene Versorgung zugänglich, angemessen und wirksam sind.

## Fazit für die Praxis

- Die konvergierenden Befunde aus Epidemiologie, kognitiven Neurowissenschaften und klinischer Forschung weisen auf die zentrale Bedeutung des Jugend- und jungen Erwachsenenalters für die Entwicklung, Behandlung und Prävention psychischer Erkrankungen hin, mit weitreichenden Konsequenzen für Forschungsfragen, dimensionalen diagnostischen Ansätzen sowie der Weiterentwicklung von klinischen Versorgungsstrukturen. Die Wirksamkeit der Früherkennung bei Psychosen und anderen psychischen Erkrankungen, wie z. B. Persönlichkeitsstörungen, sowie der große, ungedeckte klinische Bedarf bei Jugendlichen und jungen Erwachsenen sind wichtige Argumente für ein solches Vorhaben.
- Im Zusammenhang mit der weiterhin hohen Inzidenz und Krankheitslast psychischer Erkrankungen sowie den hohen Folgekosten für die Gesellschaft sind daher neue und grundlegende Überlegungen zur Forschung und Versorgung psychischen Erkrankungen dringend erforderlich. Ein Youth-Mental-Health-Paradigma für das Jugend- und junge Erwachsenenalter mit den zentralen Themen der Früherkennung und Frühbehandlung psychischer Störungen leistet einen wichtigen Beitrag, um diese dringlichen Herausforderungen zu adressieren.
